# Strength-Duration Time Constant in Peripheral Nerve: No Abnormality in Multiple Sclerosis

**DOI:** 10.1155/2012/390157

**Published:** 2012-05-08

**Authors:** Gençer Genç, Semai Bek, Tayfun Kasikci, Umit Hidir Ulas, Seref Demirkaya, Zeki Odabasi

**Affiliations:** Department of Neurology, Gulhane Medical Faculty, Etlik, Ankara 06018, Turkey

## Abstract

*Objectives*. To investigate the properties of the strength-duration time constant (SDTC) in multiple sclerosis (MS) patients. *Methods*. The SDTC and rheobase in 16 MS patients and 19 healthy controls were obtained following stimulation of the right median nerve at the wrist. *Results*. SDTC and rheobase values were 408.3 ± 60.0 *μ*s and 4.0 ± 1.8 mA in MS patients, versus 408.0 ± 62.4 *μ*s and 3.8 ± 2.1 mA in controls. The differences were not significant in SDTC or rheobase values between the patients and controls (*P* = 0.988 for SDTC and *P* = 0.722 for rheobase). *Conclusion*. Our study showed no abnormality in relapsing remitting MS patients in terms of SDTC, which gives some indirect information about peripheral Na^+^ channel function. This may indicate that alterations in the Na^+^ channel pattern in central nervous system (CNS) couldnot be shown in the peripheral nervous system (PNS) in the MS patients by SDTC. The opinion that MS can be a kind of channelopathy might be proven by performing other axonal excitability tests or SDTC in progressive forms of MS.

## 1. Introduction

Ion channels play an essential role in signal transmission and production of the action potentials by controlling anion and cation membrane traffic. These channels are important for normal functioning of the excitable tissues of the nervous system. Molecular genetics had shown that diseases due to gene encoding mutations in the ion channel subunits of cell membranes are channelopathies [1]. Ion channel mutations may affect whole nervous system. An increasing number of neurological channelopathies in CNS, PNS, and muscles have been identified and have helped to learn the molecules and cellular processes that underlie electrical excitability and disorders of excitability [2].

Although channelopathies are often inherited, those associated with autoimmune mechanisms have also been recently described [1]. Apart from this, channelopathies resulting from the aberrant transcription of a normal gene are named transcriptional channelopathies. Peripheral nerve injury and multiple sclerosis can lead to altered transcription [3].

It has not been proven that multiple sclerosis (MS) is a channelopathy. Some researchers have proposed that MS can affect the PNS in addition to the CNS [4–8]. 

Axonal excitability tests provide information about the activity of ion channels, energy-dependent pumps, and ion exchange processes activated during impulse conduction in peripheral axons. These tests are applied to the study of the biophysical properties of human peripheral nerves in vivo and give important information about axonal ion channel function and also give limited information about the underlying pathophysiological mechanisms in various neurological disorders [9, 10]. These measurements are based on the membrane potentials and other biophysical characteristics of the axons. The strength-duration time constant (SDTC) is used in nerve excitability studies and is interpreted as a measure of axonal excitability that is dependent upon the biophysical properties of the axonal membrane at the node of Ranvier. It also provides information about Na^+^ channel functioning [10, 11]. 

Specific sodium channel isoforms play an important role in the pathophysiology of MS. They take part in the restoration of impulse conduction after demyelination, axonal degeneration, and the mistuning of Purkinje neurons that leads to cerebellar dysfunction [12, 13].

Although CNS-expressed sodium channels also occur in peripheral nerves, several additional channels occur mainly in dorsal root ganglion cells. SCN9A gene encodes the *α* subunit of Nav1.7, which occurs in a subset of dorsal root ganglion neurons, as well as in sympathetic ganglia. Missense mutations cause primary erythermalgia, paroxysmal extreme pain disorder, and insensitivity to pain [14].

In a recent study, it was found that the expression of acid-sensing ion channel 1 had been associated with axonal damage in animals. Authors suggested that blockade of acid-sensing ion channel 1 had the potential to provide neuroprotective benefits in MS [15]. In another study evaluating the sodium channel expression in human astrocytes, it was suggested that the upregulated expression of Nav1.5 in astrocytes may support sodium/potassium pump-dependent ionic homoeostasis in areas of central nervous system injury [16]. Another study revealed that remyelination of dorsal column axons by endogenous Schwann cells restored the normal pattern of sodium and potassium channels at nodes of Ranvier. These channels might be possible therapeutic targets in future [17].

Although MS is a disease of the CNS, recently peripheral nerve involvement has also been proposed. The present study aimed to investigate the properties of the SDTC in MS patients.

## 2. Materials and Methods

### 2.1. Participants

The study included 20 patients (13 females, 7 males) with relapsing-remitting MS, and 20 gender- and age-matched healthy controls (13 females, 7 males). The patients were definitively diagnosed with MS according to the criteria of Poser and McDonald [18, 19]. Clinical severity was evaluated using the Expanded Disability Status Scale (EDSS) [20]. Patients with comorbid autoimmune or neoplastic pathologies, peripheral nerve disease, and systemic/metabolic disease were excluded from the study. In all, 4 MS patients and 1 control were unable to complete the study because of intolerance to electrophysiological testing. As such, the study was conducted with 16 MS patients and 19 volunteers. All patients gave their personal informed consent for the study. The study was approved by the Local Ethics Committee.

### 2.2. EMG Studies and Formulation

All the electrophysiological tests were performed with a 4-channel electromyography (EMG) machine (Dantec Keypoint, Dantec Dynamics, Bristol, United Kingdom). The SDTC, which is partially dependent upon persistent sodium conductance activity at the resting membrane potential, was measured from the median motor axon. Systemic/neurological examination and upper and lower extremity motor and sensory nerve conduction studies were performed prior to SDTC measurement.

**Figure 1:**
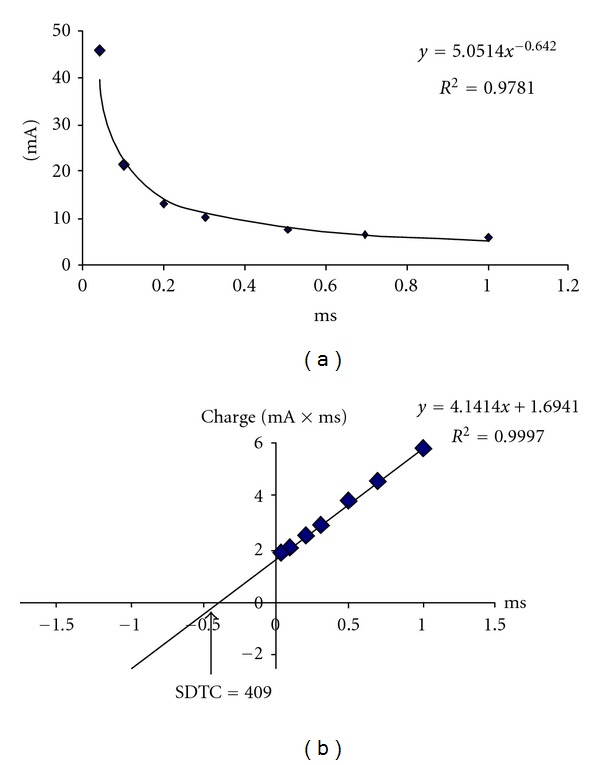
(a) The relationship between stimulus strength (mA) and stimulus duration (ms) in patient 11. (b) The relationship between stimulus duration and the stimulus charge in the same patient. The SDTC is given by the intercept of the linear regression line on the duration axis and was calculated as follows: when *y* is accepted as zero, *x* is obtained by dividing 4.1414 by 1.6941, as an example.

Right median nerve stimulation was applied at the wrist, and recordings were made at the abductor pollicis brevis (APB) muscle in order to obtain compound muscle action potentials (CMAPs). Skin temperature was monitored close to the stimulation site and kept at more than 32°C by placing a blanket over the palm and using radiant heat if necessary. The amplitude of the CMAP was measured from baseline to negative peak, and the target CMAP was set to 40% of the peak response 1 ms in duration. The stimulus strength that produced the target response for different stimulus durations (0.04, 0.1, 0.2, 0.3, 0.5, 0.7, and 1 ms) was recorded. Data were transferred to a computer to obtain stimulus-response curves showing the relationship between stimulus strength and stimulus duration (Figure 1(a)). The SDTCs were calculated using Weiss's formula [21]. The stimulus charge was obtained by multiplying the stimulus strength by stimulus duration. There was a linear relationship between the stimulus charge and stimulus duration (Figure 1(b)). Based on the regression equation for this linear relationship, the SDTC was calculated [22]. The SDTC is the point that is the intercept of the regression line on the *x*-axis (duration axis); the rheobase is given by the slope of the regression line [23]. The evaluator was blind during evaluation of the patients/controls.

### 2.3. Statistical Analysis

All data were analyzed using SPSS v.15.0 statistical software. To compare the SDTC, rheobase, and CMAP values statistically, the *t* test was used. Quantitative data are presented as mean ± standard deviation (SD). A *P*  value <0.05 was considered statistically significant.

## 3. Results

We examined 16 MS patients (12 females, 4 males) and 19 gender- and age-matched healthy controls (12 females, 7 males). Mean age of the MS patients was 31.44 ± 6.3 years, versus 32.74 ± 7.2 years for the controls. Significant differences in gender and age were not observed between the patients and controls (*P* = 0.580 for age). The mean number of annual relapses in the MS patients was 3.06 ± 1.38, mean disease duration was 5.31 ± 2.75 years, and mean EDSS score was 1.68 ± 0.79. Clinical characteristics of the MS patients are shown in Table 1.

According to the electrophysiological test results, none of the participants had polyneuropathy. Mean SDTC and rheobase values were 408.3 ± 60.0 *μ*s and 4.0 ± 1.8 mA in the MS patients and 408.0 ± 62.4 *μ*s and 3.8 ± 2.1 mA in controls, respectively. There were not any significant differences in SDTC, rheobase, or CMAP values between the patients and controls (*P* = 0.988,  *P* = 0.722, and *P* = 0.644, resp.).

## 4. Discussion

Persistent Na^+^ channels in human motor axons are those that are active at the resting membrane potential. The SDTC is dependent upon the membrane potential and is partially affected by persistent Na^+^ conductance [24]. Increasing the fraction of Na^+^ current that is persistent (or depolarizing the node) produces a longer SDTC and lower rheobase [10]. It has been shown that the SDTC was longer in patients with diseases that affect the lower motor neurons/axons [23, 25]. It has also been suggested that an increase in persistent Na^+^ channel expression associated with axonal regeneration is responsible for this phenomenon. Nerve regeneration following axonal loss results in increase in the rate of persistent Na^+^ channels, persistent Na^+^ conduction, and the SDTC.

 Multiple sclerosis is a chronic, demyelinating (also accompanied by axonal destruction) disease of the CNS, with an unknown etiology. Damage to myelinated fibers causes defective impulse transmission. Myelinated axons have more Na^+^ channels, which play a critical role in impulse conduction. Some alterations in the Na^+^ channel pattern may occur in myelinated fibers affected by MS [13]. In addition, it has been shown that the number of Na^+^ channels increases in the demyelinating lesions of MS. It has also been proposed that these channels provide Na^+^ input to neurons, trigger calcium ions in cells, and ultimately produce the axonal injury in such neuroinflammatory diseases as MS. It has also been reported that abnormal expression of Na^+^ channels contributes to the emergence of symptoms of MS [26–28]. As such, all data regarding the number, subtype, and distribution of Na^+^ channels might lead to a greater understanding of the etiology and pathophysiology of MS. It has been shown that alterations in the expression pattern of specific Na^+^ channel isoforms are important in remission and progression of MS. By manipulating these channels, it might be possible to develop new therapies for MS [13]. Currently, some trials based on the assumption that Na^+^ channel blockers may be potential neuroprotective agents in MS are being conducted. A recent study suggested that loss of Na^+^ channel *β*2 subunit is neuroprotective in EAE by prevention of Na^+^ channel upregulation in response to demyelination [29]. As recent studies showed that voltage-gated Na^+^ channels are neuroprotective in experimental models of inflammatory demyelinating disease, some Na^+^ channel blockers have been used for MS. A recent study that lamotrigine was used for neuroprotection in secondary progressive multiple sclerosis revealed that sodium-channel blockade had not prevented cerebral volume loss [30].

Since MS is primarily a disease of the CNS, studies investigating the relationship between Na^+^ channels and MS are limited by the functions of Na^+^ channels in the CNS. However, the literature contains a few studies reporting the PNS involvement in MS. In these studies segmental demyelination, reduced myelin thickness, hypertrophic neuropathy, prolonged distal latency, decreased amplitude and conduction velocity, and increased jitter have been observed [31–36]. The role of Na^+^ channels has not been evaluated since. In the present study, peripheral nerve involvement in the MS patients was investigated with SDTC. No significant difference was found between MS patients and the control group; however, the possible role of peripheral intranodal Na^+^ channels in the pathogenesis of MS was evaluated.

Involvement of the PNS was proposed to be mild and progressive in MS patients, but no significant difference was shown at the beginning of the study [7]. Although this study was investigating the temperature effects on standard nerve conduction properties but not excitability, the results of the study regarding progressive peripheral nerve involvement in MS patients differ from the present study's results. The fact that the present study included a small number of patients, and that disability in MS patients increases over time with sensory and/or motor deficits in the extremities should be taken into consideration. A prospective, investigator-blinded study that evaluated CMAP amplitudes in 4 different motor nerves in 69 MS patients reported significant lower mean CMAP amplitudes in patients than in the controls [37].

The present study examined changes in the SDTC of the median nerve in relapsing-remitting MS patients to evaluate impulse conduction disturbances in the peripheral axons and investigated the properties of the SDTC in MS patients by comparing them with those of gender- and age-matched healthy controls. Significant differences in SDTC, rheobase, and CMAP values between the study and control groups were not observed.

Contrary to Vogt's findings, the present study showed no significant difference between the CMAP amplitudes in the MS patients and controls (15.0 ± 4.3 versus 14.4 ± 3.5, resp.; *P* = 0.644). This might have been due to the small number of patients in the present study and that they had markedly lower EDSS scores (4.4 ± 0.2 versus 1.68 ± 0.79).

Considering that the SDTC increased due to an increase in the persistent Na^+^channel expression associated with nerve regeneration after axonal injury, the present study is important because it evaluated the status of Na^+^ channels and peripheral nerve involvement in MS patients. It has been shown that the SDTC was longer in patients with diseases that affect the lower motor neurons/axons, such as amyotrophic lateral sclerosis, spinal muscular atrophy, and peripheral axonal neuropathies [23, 25]. In a recent study lower motor neuron loss in MS was demonstrated electrophysiologically and morphologically [37].

The literature contains a few studies that have evaluated axonal excitability in MS patients [5, 6, 8, 38, 39]; however, the SDTC was evaluated in only 1 of these studies [39]. The other studies assessed other axonal excitability measurements, such as refractoriness, supernormality, and threshold tracking [5, 6, 8, 38]. Some studies have shown that supernormality was reduced in the peripheral nerves of MS patients [5, 6, 8]. These studies suggest that paranodal demyelination reduces supernormality due to leakage of the stored currents [10, 24]. The results of these studies differ from those of the present study. The cause of this discrepancy is usage of axonal excitability measurements other than the STDC. The potential dependence of supernormality depends primarily on the paranodal K^+^ channels, whereas the SDTC reflects the properties of persistent Na^+^ currents [10].

In another study [38] based on threshold tracking as an axonal excitability measurement in median motor axons and superficial radial sensory axons it was reported that supernormality and threshold electrotonus at the tested sites (median motor axons at the wrist, and radial sensory axons at the mid-forearm) were similar in the control and MS groups, in contrast to the studies that reported that MS patients have smaller supernormality than normal patients [5, 6, 8]. These results are similar with those of the present study. Additionally, Misawa et al. reported that approximately 5% of 60 MS patients developed demyelinating polyneuropathy [38]. Although the cause of the discrepancy between studies reporting that supernormality was reduced in the motor nerves in MS patients and those reporting it was not remains unclear, it has been suggested that threshold tracking is more accurate and sensitive for evaluating axonal excitability than amplitude tracking [24]. Additionally, it has been proposed that reduced supernormality in previous studies might be related to secondary changes in paralyzed limbs caused by MS lesions.

A recent study that included 12 MS patients and 50 healthy controls used the recovery cycle (relative refractory period, refractoriness at 2 ms, supernormality, late subnormality), threshold electrotonus to ±40% currents, and the current-threshold (I/V) relationship, as well as the SDTC, to evaluate axonal excitability [39]. The results show that there were significant differences in supernormality, late sub-normality, threshold electrotonus to ±40% currents (slow accommodation to depolarization, depolarizing threshold at 90–100 ms, depolarizing threshold undershoot), and the current-threshold (I/V) relationship (threshold to 50% depolarizing current, resting I/V slope, depolarizing I/V slope) between the 2 groups. MS patients had changes in physiological measures of axonal excitability attributable to increased slow K^+^ channel activity, indicating that these changes represent a transcriptional channelopathy due to up-regulation of K^+^ channel expression. This study shows that SDTC was identical in the 2 groups (*P* = 0.6331), is the only study in the literature that assessed the SDTC in MS patients, and resembles the present study in terms of persistent Na^+^ channel findings.

The present study was designed based on the assumption that Na^+^ channel variations that have been reported in the CNS of MS patients might also occur in the PNS. Axonal membrane excitability was evaluated using the SDTC, which is an indicator of Na^+^ channel functioning in peripheral nerve Ranvier nodes. The data presented suggest that there were no differences between the MS patients and healthy controls in terms of SDTC.

## 5. Conclusion

Strength duration time constant gives indirect information about Na^+^channels. Thus, it might not be correct to consider that Na^+^ channels in peripheral nerves of MS are completely unaffected according to our study. The absence of significant difference in peripheral intranodal Na^+^ channel functioning between the MS patients and controls in the present study indicates that alterations in the Na^+^ channel pattern, which have been shown in the CNS of MS patients, could not be shown in PNS of MS patients by SDTC. As such, we suggest that CNS pathologies are fundamentally involved in the pathogenesis of MS, and even if the PNS is affected this might occur mostly during the later stages of the disease. The opinion that MS is a channelopathy might be proven by performing other axonal excitability tests or SDTC in progressive forms of MS. Although persistent Na^+^ channels do not appear to play a role in this process, additional prospective studies (including large number of patients) that evaluate the properties of the SDTC in MS patients are needed in order to gather more evidence.

## Figures and Tables

**Table 1:** Clinical characteristics of MS patients.

Patient	Age	Number of relapses	Disease duration (year)	EDSS
1	34	2	3	1
2	30	3	10	1,5
3	25	2	3	1
4	29	3	5	2
5	24	2	4	1
6	37	2	5	1
7	25	2	3	1
8	31	4	12	2
9	22	3	3	2
10	39	4	8	2,5
11	37	2	4	1
12	31	3	3	2
13	26	7	8	3,5
14	30	2	4	1
15	40	3	5	1,5
16	43	5	5	3
